# Remyelination Is Correlated with Regulatory T Cell Induction Following Human Embryoid Body-Derived Neural Precursor Cell Transplantation in a Viral Model of Multiple Sclerosis

**DOI:** 10.1371/journal.pone.0157620

**Published:** 2016-06-16

**Authors:** Warren C. Plaisted, Angel Zavala, Edna Hingco, Ha Tran, Ronald Coleman, Thomas E. Lane, Jeanne F. Loring, Craig M. Walsh

**Affiliations:** 1 Department of Molecular Biology & Biochemistry, Sue and Bill Gross Stem Cell Center, Multiple Sclerosis Research Center, Institute for Immunology, University of California Irvine, Irvine, California, United States of America; 2 Center for Regenerative Medicine, Department of Chemical Physiology, The Scripps Research Institute, La Jolla, California, United States of America; 3 Department of Pathology, University of Utah, School of Medicine, Salt Lake City, Utah, United States of America; University of Pennsylvania, UNITED STATES

## Abstract

We have recently described sustained clinical recovery associated with dampened neuroinflammation and remyelination following transplantation of neural precursor cells (NPCs) derived from human embryonic stem cells (hESCs) in a viral model of the human demyelinating disease multiple sclerosis. The hNPCs used in that study were derived by a novel direct differentiation method (direct differentiation, DD-NPCs) that resulted in a unique gene expression pattern when compared to hNPCs derived by conventional methods. Since the therapeutic potential of human NPCs may differ greatly depending on the method of derivation and culture, we wanted to determine whether NPCs differentiated using conventional methods would be similarly effective in improving clinical outcome under neuroinflammatory demyelinating conditions. For the current study, we utilized hNPCs differentiated from a human induced pluripotent cell line via an embryoid body intermediate stage (EB-NPCs). Intraspinal transplantation of EB-NPCs into mice infected with the neurotropic JHM strain of mouse hepatitis virus (JHMV) resulted in decreased accumulation of CD4+ T cells in the central nervous system that was concomitant with reduced demyelination at the site of injection. Dampened neuroinflammation and remyelination was correlated with a transient increase in CD4+FOXP3+ regulatory T cells (Tregs) concentrated within the peripheral lymphatics. However, compared to our earlier study, pathological improvements were modest and did not result in significant clinical recovery. We conclude that the genetic signature of NPCs is critical to their effectiveness in this model of viral-induced neurologic disease. These comparisons will be useful for understanding what factors are critical for the sustained clinical improvement.

## Introduction

Multiple sclerosis (MS) is considered a chronic autoimmune disorder affecting the central nervous system (CNS) in which infiltration and accumulation of lymphocytes in the brain and spinal cord leads to demyelination followed by axonal degeneration. Early stages of the disease are characterized by transient inflammation and compensatory remyelination resulting in a cycle of descending neurologic dysfunction and limited recovery [1, 2]. However, endogenous myelin repair is not sustainable and ultimately gives way to a stage of chronic neurodegeneration and progressive accumulation of disability. Current FDA-approved disease-modifying therapies (DMTs) target the immune component of MS and have demonstrated effectiveness in reducing relapse rates, although this is often not sustainable [3]. However, the most commonly prescribed DMTs do not directly address white matter damage in the CNS and are consequently ineffective at treating advanced stages of MS. Therefore, there remains an unmet need for a treatment strategy that addresses inflammatory cell infiltration while promoting long-term remyelination.

Neural precursor cells (NPCs) have emerged as a viable therapeutic target for the treatment of a variety of neurological disorders. Previously, transplantation of NPCs was shown to attenuate disease pathology in animal models of Alzheimer’s disease, Parkinson’s disease, Huntington’s disease, and spinal cord injury [4–8]. Human NPCs have also been shown to differentiate into myelin-competent oligodendrocytes and directly remyelinate host axons when transplanted into the CNS of hypomyelinated mice [9]. Importantly, in rodent and non-human primate models of MS where neuroinflammatory demyelination is triggered via immunization against myelin peptides, NPCs suppress encephalitogenic T cell activation and enhance endogenous myelin regeneration [10–13].

We have recently shown that intraspinal injection of NPCs derived from human embryonic stem cells (hESCs) using a direct differentiation method (DD-NPCs) promotes neurologic recovery in mice afflicted with virus-induced immune-mediated demyelination [14]. Importantly, recovery was facilitated by the emergence of immunosuppressive CD4+/CD25+/FOXP3+ regulatory T cells (Tregs) and was not dependent on long-term engraftment of the human NPCs. Thus, human pluripotent stem cells (PSCs) may serve as an inexhaustible source from which to generate NPCs for the treatment of MS. For clinical cell therapy, induced pluripotent stem cells (iPSCs) may be preferable to hESCs, because they retain the genetic background of the donor, which may circumvent the requirement for immunosuppressant drugs that leave patients vulnerable to infection, tumor progression and re-activation of dormant viruses.

Previous work with neural cell types has shown that variations in derivation and culture protocols can lead to NPCs with distinctly unique gene expression profiles [15]. Here we demonstrate that NPCs generated using a differentiation methodology that includes an embryoid body stage produce cells (EB-NPCs) with a unique gene expression profile and diminished clinical effect than the DD-NPCs derived by our previously published method. EB-NPC transplanted mice did not show a significant clinical improvement, but did exhibit dampened accumulation of CD4+ T cells in the CNS. Reduced T cell infiltration was correlated with focal remyelination and a transient increase in CD4+/FOXP3+ Tregs in draining cervical lymph nodes (CLNs). EB-NPCs induced the differentiation of naïve CD4+ T cells into Tregs *in vitro*, and *in vivo* ablation of Tregs abrogated EB-NPC-induced histopathological improvement. These findings show that the gene expression profiles of hNPCs derived by different methods correlate with clinical effectiveness of the cells in a model of MS. Despite these differences, hNPCs derived using these distinct methods similarly promote the generation of anti-inflammatory Tregs, and assist in focal remyelination. These comparisons provide initial information about what specific characteristics make hNPCs effective for treatment of inflammation and demyelination in MS.

## Materials and Methods

### Derivation and Maintenance of EB-NPCs

The human iPSC line HDF51iPS1 was reprogrammed from primary fetal fibroblasts as previously described [16]. hiPSCs were adapted to feeder-free conditions and maintained in Essential 8 medium (Thermo Fisher) on Geltrex-coated (Thermo Fisher) dishes. For lentiviral transduction of the luciferase expression cassette, the pLenti CMV Puro LUC plasmid (Addgene) was packaged and viral particles collected as described in [14]. Once hiPSCs reached approximately 50% confluence in one well of a 6-well dish, viral supernatant was added to hiPSCs in culture with 10 μg/ml polybrene (Sigma). Cells were spun in the dish at 400RCF for 1 h at 37°C. After 48 h, 1 μg/ml puromycin was added to select for hiPSCs stably expressing luciferase. Transduced hiPSCs were maintained in 1 μg/ml puromycin and differentiated to NPCs according to established methods [17]. Briefly, feeder-free hiPSCs were dissociated using 0.5 mM EDTA (Thermo Fisher), transferred to ulta-low adherence 6-well plates, and cultured for 5 days in human ESC medium (DMEM/F12 + GlutaMAX, 20% Knockout Serum Replacement, 1X non-essential amino acids, and 0.1 mM 2-mercaptoethanol; all from Thermo Fisher) supplemented with 500 ng/ml recombinant Noggin (R&D Systems) and 10 μM SB431542 (Tocris). On the fifth day of culture in hESC medium, hiPSCs formed embryoid body-like structures (EBs) and 20–50 EBs were transferred to each well of a Geltrex-coated 6-well dish. Increasing amounts of N2 medium (DMEM/F12 + GlutaMAX, 1X N2 supplement [Thermo Fisher]) supplemented with 500 ng/ml Noggin and 10 μm SB431542 were added every other day for 14–16 days. Resulting columnar rosette structures were collected using Accutase (BD) and labeled with anti-CD184, anti-CD24, anti-CD44, and anti-CD271 antibodies according to manufacturer specifications using the BD Stemflow Human Neural Cell Sorting Kit. Sorted EB-NPCs were maintained in NPC medium (DMEM/F12 + GlutaMAX, 0.5X N2, 0.5X B27 without vitamin A [Thermo Fisher], 20 ng/ml bFGF [Thermo Fisher]) on Geltrex-coated dishes and passaged using Accutase when cell density reached 80–90% confluence.

### Characterization of EB-NPCs

For flow cytometric analysis of EB-NPCs, cells were collected using Accutase and stained with mouse anti-Nestin, mouse anti-human Sox1, and mouse anti-Sox2 per manufacturer’s instructions using the Human Neural Lineage Analysis kit (BD). For immunofluorescence microscopy, EB-NPCs were seeded on Geltrex-coated slides, fixed with 4% paraformaldehyde, and stained with rat anti-Nestin (1:500; Millipore), rabbit anti-Sox2 (1:200; Epitomics), or rabbit anti-Pax 6 (1:50; BioLegend) before addition of respective secondary antibodies (goat anti-rabbit AlexaFluor 568 and goat anti-rat AlexaFluor 488; both from Thermo Fisher). For analysis of multipotency, EB-NPCs were cultured in neuronal differentiation medium (Neurobasal, 1X B27, 1X GlutaMAX), astrocyte differentiation medium (DMEM [Thermo Fisher], 1X N2, 1X GlutaMAX, 1% FBS [Atlanta Biologicals]), or oligodendrocyte differentiation medium (Neurobasal, 1X B27, GlutaMAX, 30 ng/ml T3 [Sigma]) for at least 14 days before being fixed with 4% paraformaldehyde and stained with mouse anti-beta-III-tubulin (1:500; Abcam), rabbit anti-GFAP (1:200; Thermo Fisher), rabbit anti-NG2 (1:200; Chemicon), or rabbit anti-Olig2 (1:100; Abcam). Slides were cover-slipped and mounted using VectaShield Hard Set Mounting Medium with DAPI (Vector Labs), and all images were captured using a Nikon Eclipse Ti inverted microscope.

### Whole genome analysis

For iNPC, eNPC and PSC samples, one well of a 6-well plate containing approximately 10^6 cells was collected and pelleted by centrifugation. Each pellet was flash frozen and stored at -80°C. EBNPCs were frozen in RNAlater (Thermo Fisher Scientific). RNA was purified using the mirVana miRNA Isolation Kit (Thermo Fisher Scientific). Collected RNA was quantified using the Qubit Fluorometer and RNA BR assay kit (Thermo Fisher Scientific). The RNA quality of each sample was determined using the 2100 Bioanalyzer (Agilent Technologies) to obtain an RNA integrity Number (RIN). Samples with acceptable RINs (9.0–10.0) were amplified, labeled and hybridized onto Illumina HumanHT-12 v4 Expression Beadchips according to the manufacturer's instructions. Hybridized chips were scanned using the iScan system (Illumina). Raw data extraction was performed with Genome Studio (Illumina) and probes without a detection p-value (a measure of confidence that the signal observed is above background fluorescence) of less than 0.01 in at least 1 sample were removed. The remaining probes were then quantile-normalized to correct for between-sample variation. Normalized data were then analyzed using Qlucore Omics Explorer (Qlucore). The gene expression array data are available at the NCBI GEO database under the accession designation GSE81910.

### Animal Care and Infection

Age-matched male C57BL/6 mice (H-2b, National Cancer Institute) were anesthetized before being infected intracranially (i.c.) with 150 plaque-forming units (p.f.u.) of the JHM (J2.2-V-1) strain of MHV in 30 μl HBSS. Following infection, mice were monitored twice daily kept in a room with a 14-hour ligh/10-hour dark cycle. In order to ensure that mobility impaired mice had sufficient access to food and water, water bottles were fitted with extended 3.5 inch spouts, fresh water-saturated food pellets were provided on cage floor twice daily, and hand fed water and/or DietGel 76A (ClearH2O). Clinical evaluation was based on the following scoring system: 0, asymptomatic; 0.5, ruffled fur; 1, limp tail; 2, waddling gait without righting difficulty; 2.5, waddling gait accompanied by righting difficulty; 3, hind-limb weakness and extreme righting difficulty; 3.5, complete hind limb paralysis; and 4, death. Mice were sacrificed via isofluorane inhalation at defined time points post-transplant for tissue harvesting and analysis. The animal protocols and procedures used for these studies were reviewed and approved by the Institutional Animal Care and Use Committee of the University of California, Irvine.

### Transplantation of EB-NPCs

Mice previously inoculated with JHMV were injected with 250,000 EB-NPCs or human fetal fibroblasts resuspended in 2.5 μl HBSS, at the T8-T9 vertebral level on day 14 post-infection (p.i.) as previously described [18]. Some JHMV-infected animals received 2.5 μl HBSS intraspinally as vehicle control.

### In vivo imaging of firefly luciferase activity

Firefly luciferase expression was verified *in vitro* by addition of D-luciferin (Caliper Life Sciences) to culture wells followed by detection of chemiluminescence at 560 nm using a Bio-Rad Gel Doc system. For *in vivo* detection of chemiluminescence, EB-NPC transplanted mice were injected with 250 mg/kg D-luciferin approximately ten minutes before imaging. Mice were then anesthetized using a sub-lethal dose of isofluorane delivered via inhalation. Bioluminescence due to firefly luciferase activity was captured using the IVIS Series Pre-clinical In Vivo Imaging System (PerkinElmer), and a pseudocolored image of bioluminescent intensity was overlaid onto a gray-scale photograph of the mice.

### Flow Cytometric Analysis of Spinal Cords

Animals were euthanized via inhalation of a lethal dose of isofluorane and cardiac perfusion with PBS was performed. Spinal cords or cervical lymph nodes were dissected into single-cell suspension, depleted of red blood cells via ammonium-chloride-potassium, and passed through a discontinuous Percoll gradient as previously described [19]. Single-cell suspensions were filtered, washed, and counted before being blocked with anti-mouse CD16/32 (1:200; BD Biosciences). Cells were subsequently stained with anti-CD4 (FITC-conjugated GK1.5; BD Biosciences), anti-CD8 (PE-Cy7-conugated Ly-2; BD Biosciences), anti-CD45 (APC-conjugated 30-F11; eBioscience), anti-F4/80 (FITC-conjugated Ci-A3-1; Serotech), anti-FOXP3 (EF660-conjugated FJK-16s; eBioscience) or PE-conjugated tetramers I-Ab/M133–147 and Db/S510–518 (8 μg/ml; National Institute of Allergy and Infectious Diseases MHC Tetramer Core Facility). Single-stain samples and IgG or IgM isotype controls were used to establish PMT voltages, gating, and compensation parameters. Cells were processed using an LSR II flow cytometer (BD) and analyzed using FlowJo software (Tree Star).

### Immunohistochemistry and analysis of histopathology

Animals were euthanized via inhalation of a lethal dose of isofluorane and cardiac perfusion with PBS was performed. Spinal cords were dissected and fixed overnight in 4% paraformaldehyde before being embedded in OCT compound. Frozen tissues were serially sectioned and stained with luxol fast blue (LFB) and counterstained with hematoxylin and eosin (H&E) to assess the severity of demyelination. The total area of the white matter was quantified and compared to the area of demyelinated regions using ImageJ software (NIH). All demyelination measurements were performed independently by two investigators. For identification of engrafted EB-NPCs, 6 um coronal spinal cord sections were stained with STEM121 (1:200; Clontech Laboratories) and a biotinylated secondary antibody (1:400; Vector Labs) before the ABC Elite staining system (Vector Labs) and diaminobenzidine (DAB; Vector Labs) were applied for visualization.

### Transmission electron microscopy

Mice were sacrificed via inhalation of a lethal dose of isofluorane and cardiac perfusion was performed using 0.1 M cacodylate buffer containing 2% paraformaldehyde and 2% glutaraldehyde. Spinal cords were dissected and embedded in EPON epoxy resin before being ultrasectioned, stained with uranyl acetate-lead citrate, and imaged using a transmission electron microscope according to standard protocols. G-ratios were determined by measuring axon diameter and comparing it to the total fiber diameter (axon diameter/total fiber diameter) using ImageJ software (National Institutes for Health). Measurements were performed independently by two investigators and at least 300 axons were measured per experimental group.

### In vivo Treg ablation

JHMV-infected mice were injected intraperitoneally (i.p.) with 150 μg of a rat monoclonal antibody specific for CD25 (rat anti-mouse CD25, clone PC61.5 [20, 21]) or control rat immunoglobulin G (Sigma) as previously described [14]. Efficiency of anti-CD25 treatment in transplanted mice was determined by collection of peripheral blood from the peri-orbital sinus of anesthetized animals, followed by quantification of the frequency of circulating Tregs by FACS.

### T cell—EB-NPC co-culture and Treg induction

Spleens were dissected from naïve age-matched C57BL/6 mice and total T cells were isolated using the EasySep Mouse T cell Isolation Kit (STEMCELL Technologies). Mitomycin C (Roche) treated EB-NPCs were mixed with isolated T cells at defined proportions in round-bottom 96-well plates and incubated at 37°C, 5% CO2 for 3 days in 200 μl final volume of complete T cell medium (RPMI-1640 [Thermo Fisher], 1X GlutaMAX-1 [Thermo Fisher], 1X non-essential amino acids [Thermo Fisher], 100 U/ml penicillin [Thermo Fisher], 100 μg/ml streptomycin [Thermo Fisher], 1 mM sodium pyruvate [Thermo Fisher], 55 μM 2-mercaptomethanol [Thermo Fisher], and 10% FBS [Atlanta Biologicals]). In wells where activation of T cells was desired, Dynabeads Mouse T-Activator CD3/CD28 beads (Life Technologies) were added at a concentration of 1 bead per T cell.

### Enzyme-linked immunosorbent assays (ELISAs)

Active TGF-β1 and TGF-β2 production by EB-NPCs and DD-NPCs was determined using human Quantikine ELISA Kits from R&D Systems in accordance with the manufacturer’s specifications.

### Statistics

Data were analyzed using Prism software (GraphPad). Unless otherwise noted, comparisons were performed using one-way analysis of variance, followed by post hoc analysis using Tukey’s procedure where appropriate.

## Results

### EB-derived neural precursor cells are rapidly rejected following transplantation into mice persistently infected with JHMV

**Fig 1:**
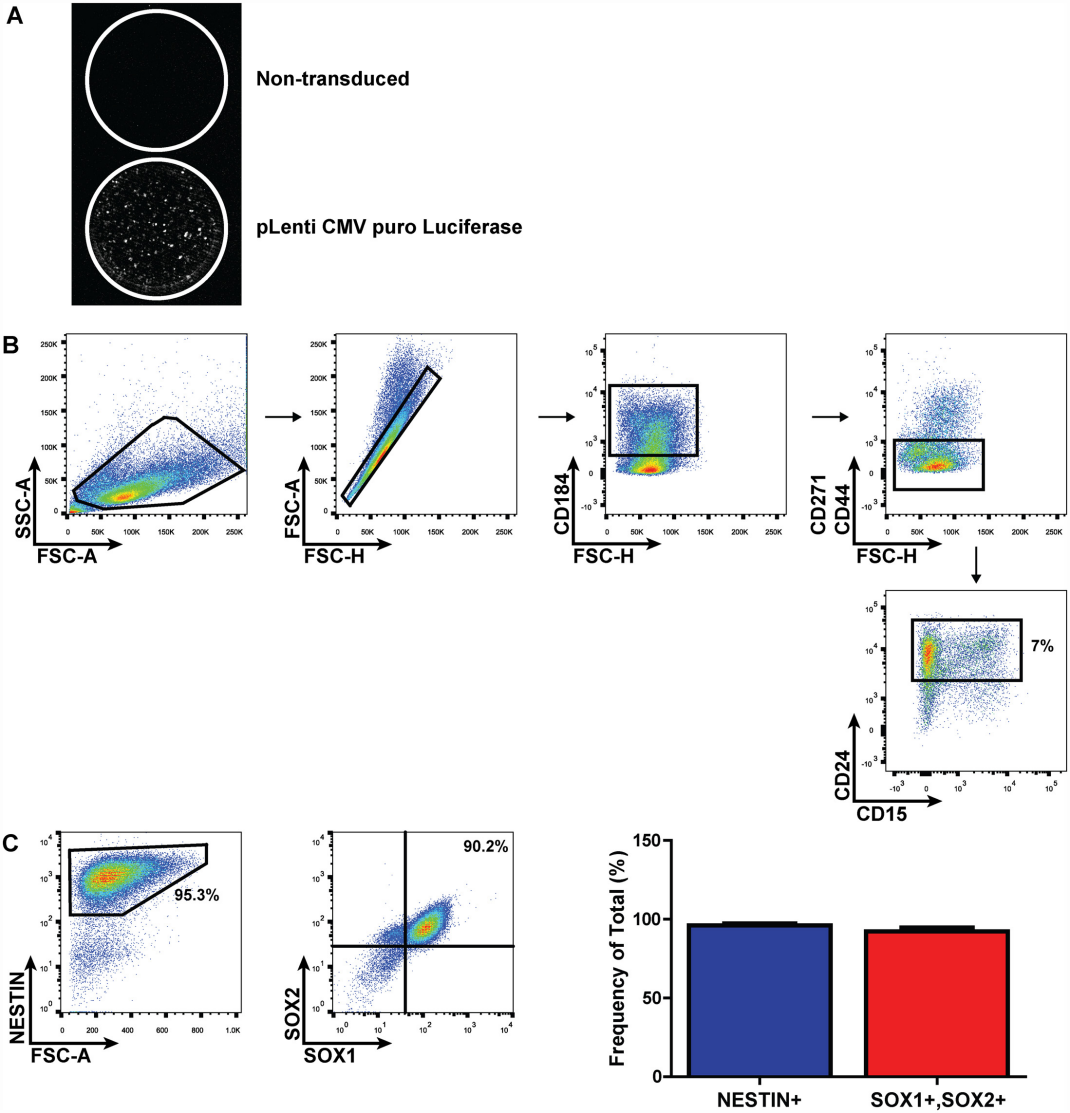
Lentivirus-transduced human iPSCs differentiate to multipotent neural precursor cells. (**A**) Representative micrographs of human iPSCs transduced with a lentiviral vector containing the *Photinus pyralis* luciferase gene and puromycin resistance cassette. Photon emission in response to D-luciferin could be detected in transduced puromycin-selected iPSCs (bottom) but not in non-transduced iPSCs (top) *in vitro*. (**B**) Representative FACS plots demonstrating CD184+/CD271-/CD44-/CD24+ gating scheme for isolation of NPCs from differentiated iPSCs. (**C**) Representative dot plots and quantification of NPC marker expression in FACS-sorted EB-NPCs following recovery and propagation (n = 3); data presented as average±SEM.

In order to evaluate graft survival and migration following intraspinal delivery of EB-NPCs, the feeder-free adapted HDF51iPS1 iPSC line was transduced with lentivirus expressing the *Photinus pyralis* firefly luciferase gene. Following puromycin selection, iPSCs constitutively expressing luciferase produced detectable photons in response to D-luciferin *in vitro* (Fig 1A). Luciferase-expressing iPSCs were differentiated to NPCs as previously described [17]. This method results in heterogeneous cultures of neural stem and precursor cells, neural crest cells, and glial cells, and hPSCs exhibit variability in their potential to differentiate to NPCs [22, 23]. Therefore, differentiated cells were enriched for NPCs via flow cytometric sorting (FACS) using the markers CD24, CD184, CD271, and CD44 (Fig 1B) [22, 24]. Isolation of CD184+/CD24+/CD271-/CD44- cells resulted in a population highly enriched for the hallmark neural stem/precursor cell marker NESTIN (96.2% ± 1.908; n = 3), and the majority of cells were double-positive for NPC markers SOX1 and SOX2 (92.3% ± 4.349%; n = 3), with some cells expressing the dorsal forebrain marker PAX6 (Fig 1C **and** S1A–S1C Fig). Furthermore, when sorted EB-NPCs were subjected to directed differentiation conditions for ≥ 14 days, markers indicative of neurons (Tuj1), astrocytes (GFAP), and oligodendrocyte lineages (NG2 and Olig2) were detected, confirming multipotency (S1D–S1G Fig). Therefore, we use the term EB-NPC to describe a Nestin+/Sox1+/Sox2+ population with the capacity to differentiate into cells expressing markers restricted to neuronal, astroglial, or oligodendroglial lineages.

**Fig 2:**
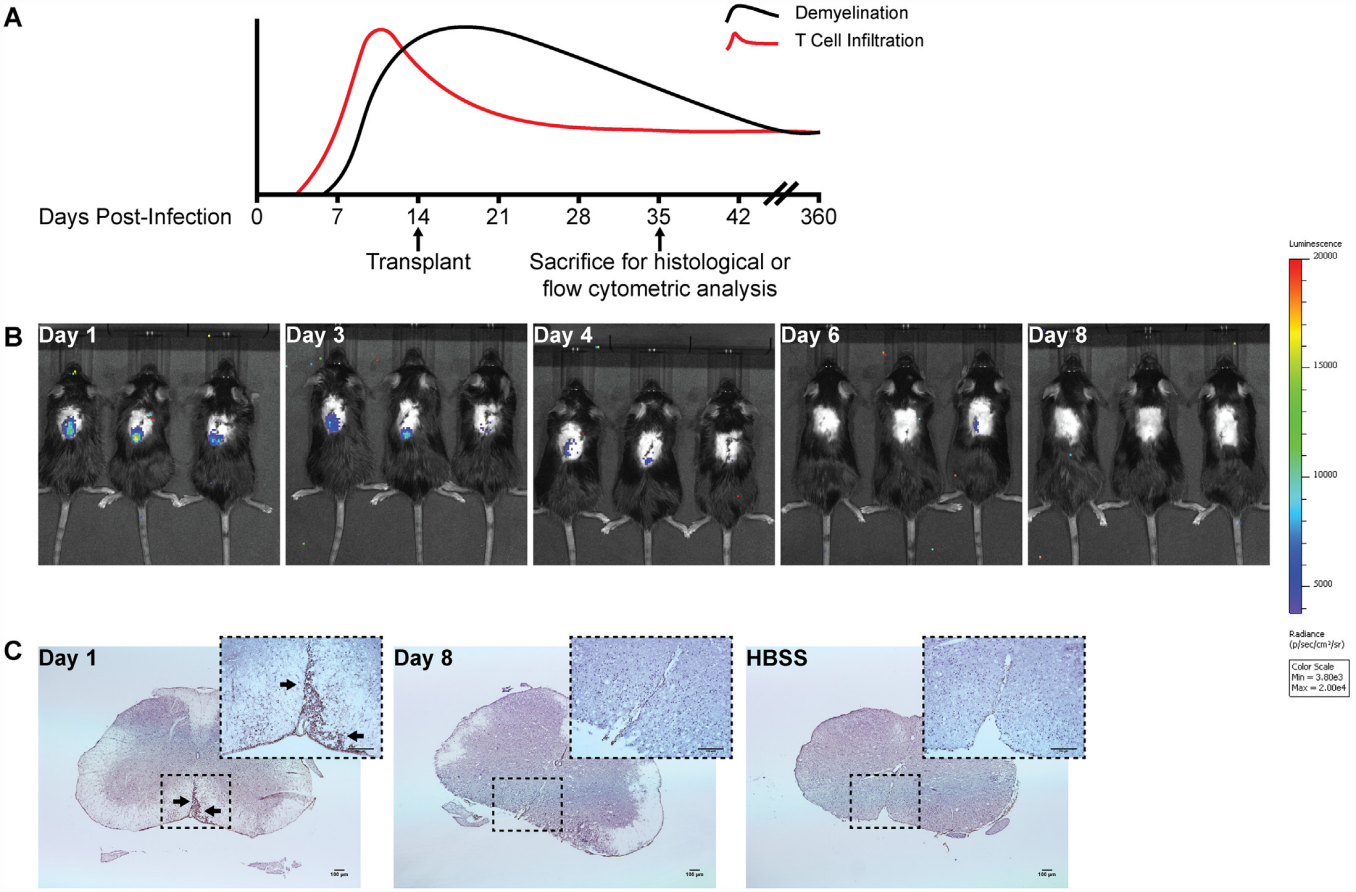
Human iPSC-derived NPCs are rapidly rejected following intraspinal transplantation in JHMV-infected mice. (**A**) Timeline highlighting the relationship of T cell infiltration and demyelination in response to JHMV infection of the CNS as well as when cells are transplanted into the spinal cord and when animals are sacrificed to assess histology and immune cell infiltration into the CNS. (**B**) *In vivo* bioluminescence imaging revealed EB-NPCs could be detected in the spinal cords of transplanted animals as early as day 1 post-transplant (p.t.) and were undetectable by day 8 pt. (**C**) Representative brightfield images of coronal spinal cord sections from EB-NPC transplanted mice stained with SC121, a monoclonal antibody specific for human cytoplasm. Arrows indicate SC121+ regions. Human cells were detected in ventral white matter regions at day 1 p.t. but could be not be detected by day 8 p.t., confirming rejection of EB-NPCs. Scale bars = 100 μm.

Mice intracranially inoculated with a neuroadapted JHM strain of mouse hepatitis virus (JHMV) develop inflammatory lesions in the white matter tracts of the brain and spinal cord, ultimately resulting in hind-limb paralysis [25]. To evaluate clinical and immunological consequences of transplantation, EB-NPCs were injected into the spinal cords of JHMV-infected immunocompetent mice at day 14 p.i. which represents a time in which chronic neuroinflammatory demyelination is established (Fig 2A) [14, 18]. *In vivo* bioluminescence (IVIS) imaging revealed that transplanted EB-NPCs were present in the spinal cords of mice at day 1 post-transplant (p.t.). However, a marked reduction in luciferase activity was detected at day 4 p.t., and by day 8 p.t., EB-NPCs were undetectable by IVIS imaging (Fig 2B). Histologic analysis of a human-specific cytoplasmic marker confirmed the loss of EB-NPCs in the spinal cords of transplanted mice, suggesting rejection of EB-NPCs was complete by approximately 1 week p.t. (Fig 2C).

### Demyelination and T cell infiltration is reduced in EB-NPC-transplanted animals

**Fig 3:**
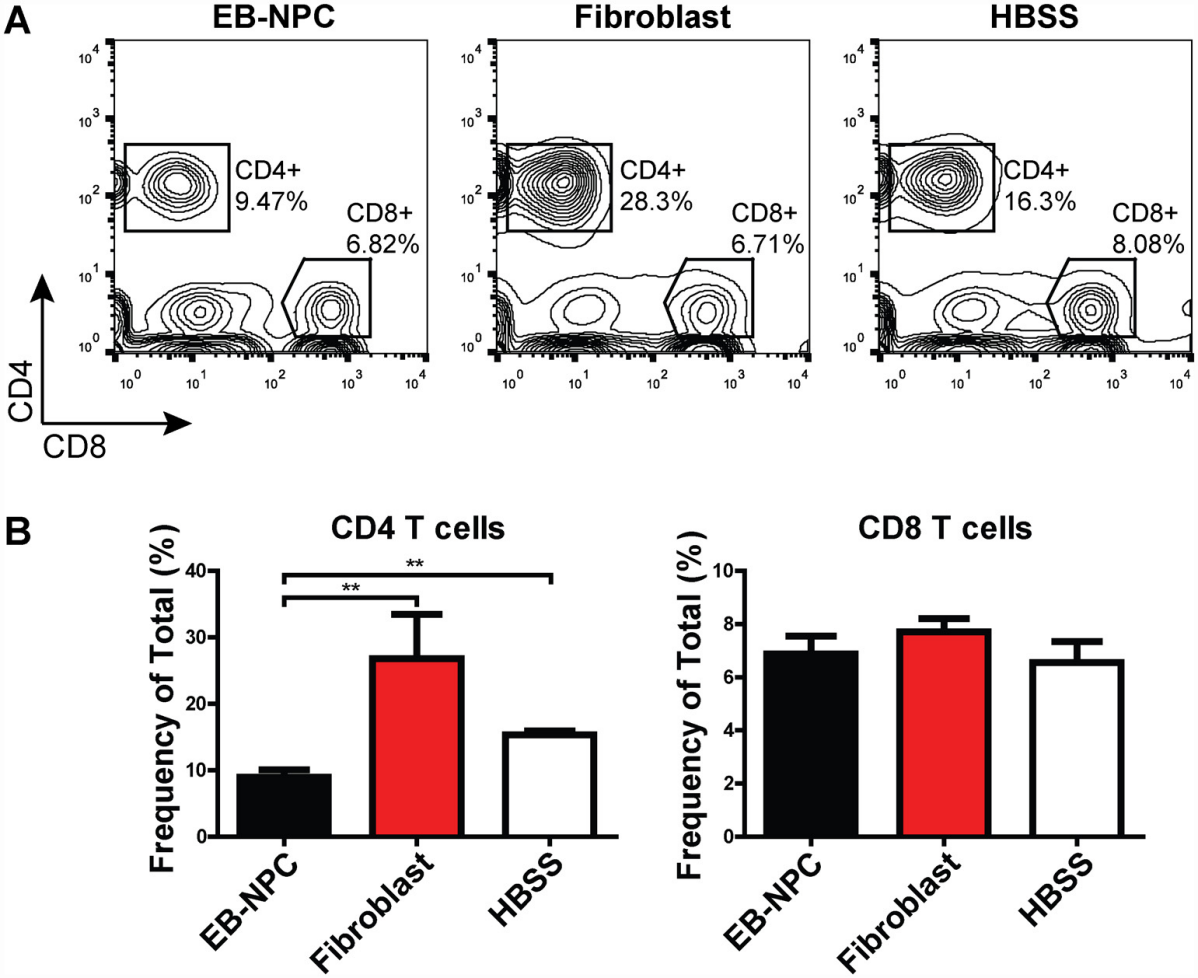
CD4 T cell accumulation is reduced in EB-NPC-transplanted mice. (**A**) Representative FACS plots demonstrating the frequency of CD4+ and CD8+ cells in the spinal cords of EB-NPC, human fibroblast, and HBSS-injected mice at day 21 pt. (**B**) Quantification of the frequency of CD4 and CD8 T cells. The frequency of CD4 T cells was significantly (p < 0.01) reduced in EB-NPC-transplanted animals compared to animals injected with human fibroblasts or HBSS. Data represents two independent experiments with n = 6 (EB-NPC), n = 4 (fibroblast), and n = 5 (HBSS) animals per group. Data presented as average+SEM and was analyzed using one-way ANOVA followed by Tukey’s multiple comparison test.

We have previously shown that animals transplanted with NPCs derived by a novel method sustained clinical recovery associated with reduced neuroinflammation and demyelination [14]. JHMV-infected animals that received an intraspinal transplant of EB-NPCs at day 14 p.i. did not exhibit clinical benefits as compared to animals transplanted with human dermal fibroblasts, or animals intraspinally injected with HBSS (S2 Fig). Flow cytometric analysis of the 12 mm spinal cord region spanning the injection site revealed a significant (p<0.01) reduction in CD4+ T cell frequency within the CNS of EB-NPC transplanted animals compared to controls, indicating dampened CD4+ T cell accumulation as a result of transient EB-NPC engraftment (Fig 3A and 3B). However, no differences in the frequencies of CD8+ T cells, virus-specific CD4+ and CD8+ T cells, macrophages, (CD45^hi^F4/80^+^) or microglia (CD45^low^F4/80^+^) were detected between HBSS, fibroblast, and EB-NPC injected animals at day 21 p.t. (Fig 3A and 3B **and** S3 Fig).

**Fig 4:**
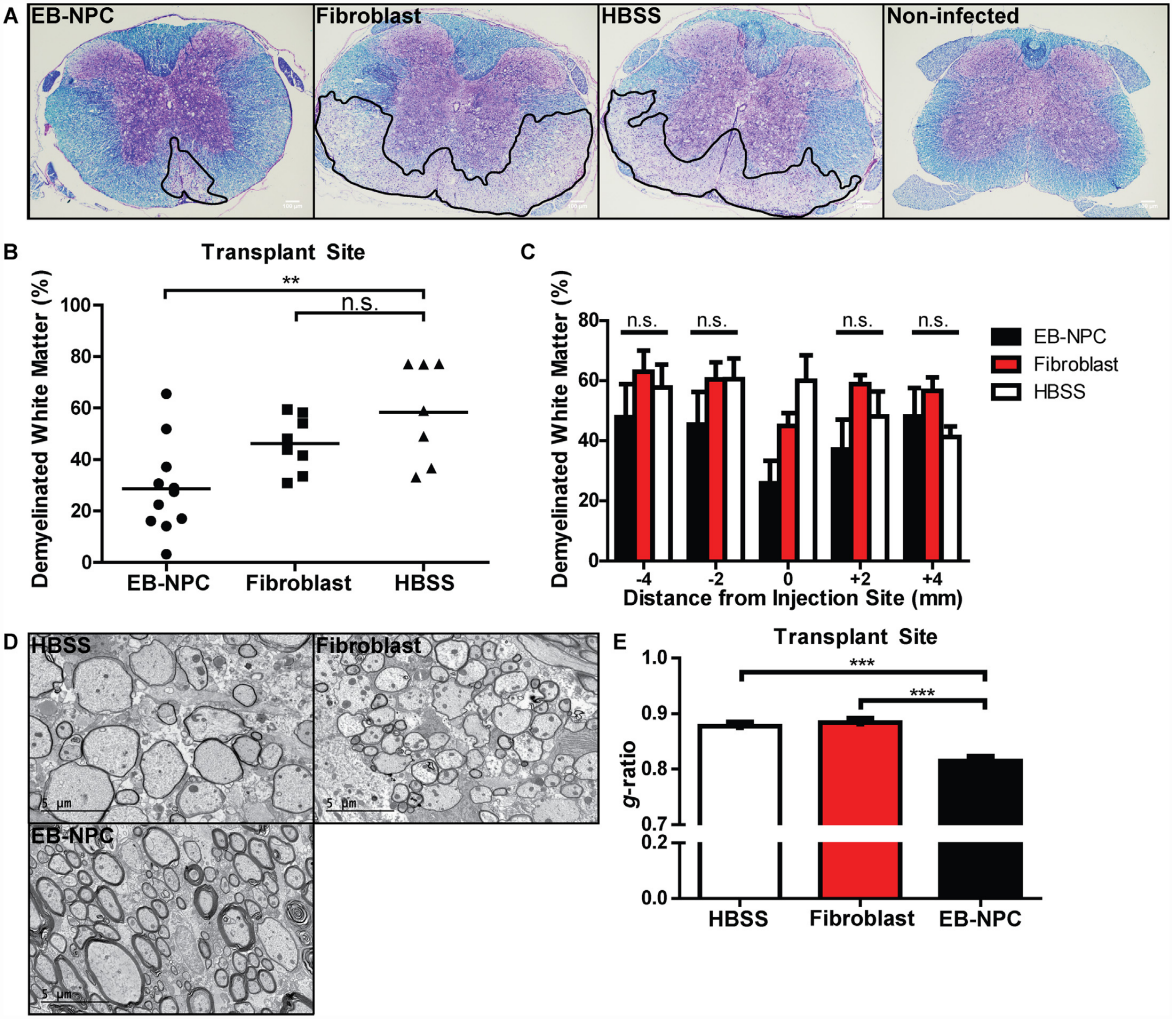
Focal remyelination in animals transplanted with EB-NPCs. (**A**) Representative brightfield images of coronal spinal cord sections stained with luxol fast blue (LFB) and counter-stained with hemotoxylin and eosin (H&E). (**B**) Quantification of demyelination in the ventral white matter of EB-NPC, fibroblast, and HBSS injected mice revealed significantly (p < 0.01) reduced demyelination at the injection site in the spinal cords of EB-NPC-transplanted mice. (**C**) Quantification of demyelination in areas adjacent to the injection site revealed that reduced demyelination was not sustained along the rostrocaudal axis. (**D**) Representative electron micrographs of coronal spinal cord sections from HBSS, fibroblast, and EB-NPC-injected mice. (**E**) Analysis of the ratio of the axon diameter vs. total fiber diameter (*g*-ratio) confirmed enhanced remyelination at the transplant site of EB-NPC-injected mice compared to controls (p < 0.001). For (***B***) and (***C***), data represents two independent experiments with n = 11 (EB-NPC), n = 8 (Fibroblast), and n = 7 (HBSS) animals per group. For (***D***), ≥ 300 axons were measured per experimental group. All data is presented as average ± SEM and was analyzed using one-way ANOVA followed by Tukey’s multiple comparison test.

Luxol fast blue (LFB) staining of serially-sectioned spinal cord tissue revealed that EB-NPC transplanted animals had reduced demyelination compared to fibroblast and HBSS injected controls (Fig 4A). Quantification confirmed a significant (p<0.01) difference in demyelination at the transplant site of EB-NPC-transplanted animals (28.6% ± 5.4%, n = 11) compared to HBSS-injected controls (58.4% ± 7.3%, n = 7) (Fig 4B). However, the observed reduction in demyelination was not sustained along the rostro-caudal axis relative to the injection site, indicating that EB-NPC sparing of myelin was focally restricted (Fig 4C). We further investigated the extent of remyelination in EB-NPC-transplanted animals by performing transmission electron microscopy (TEM) of spinal cord sections and compared the axon diameters of neurons to total myelinated fiber diameters. EB-NPC transplanted mice had extensive signs of remyelination compared to HBSS or fibroblast injected animals, and *g*-ratio analysis confirmed a significant (p<0.001) difference in myelin status at the site of transplant (Fig 4D and 4E).

### Regulatory T cells are induced following EB-NPC transplantation and are required for histopathological improvement

**Fig 5:**
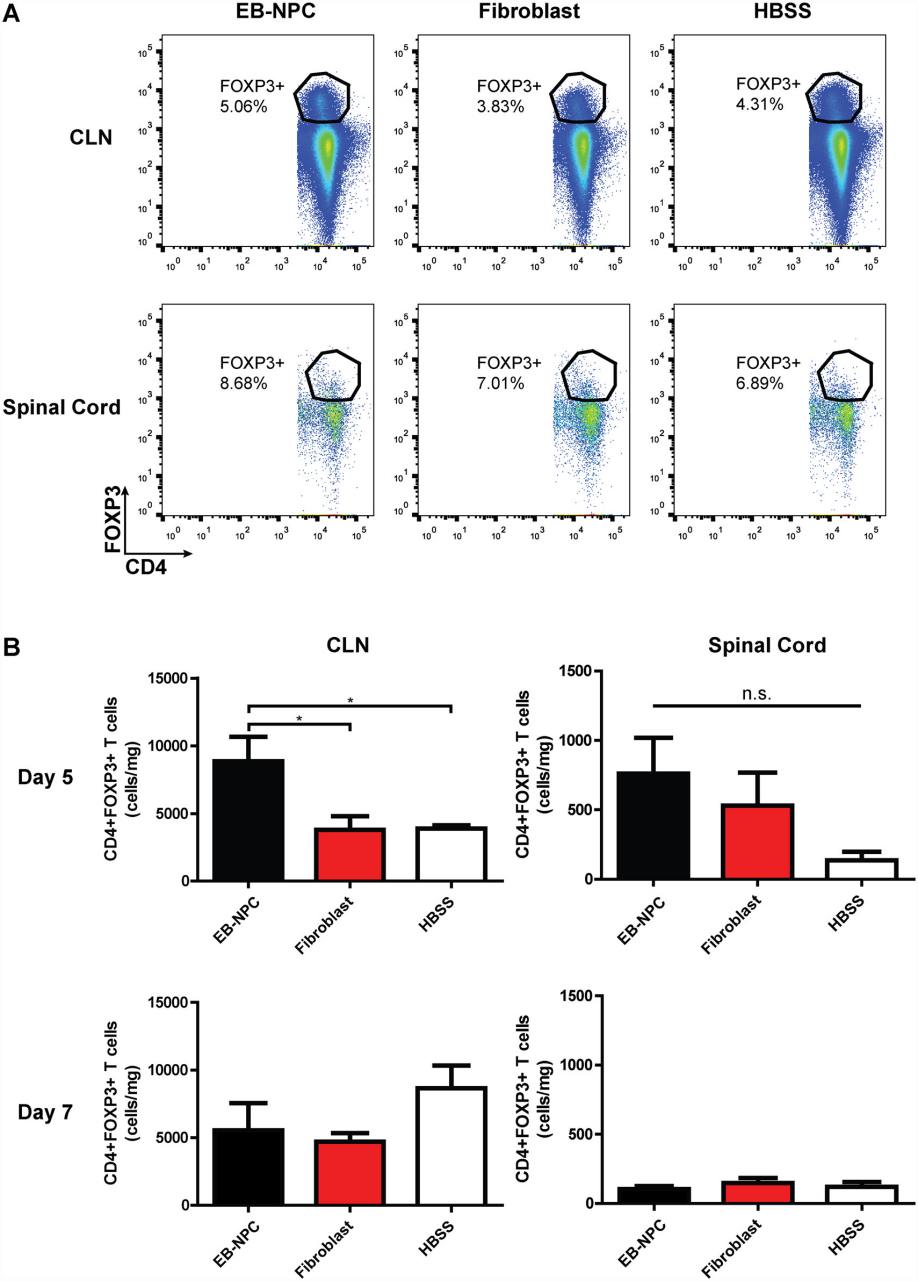
Regulatory T cells are increased in the draining cervical lymph nodes as a result of transient EB-NPC engraftment. (**A**) Representative FACS plots of CD4+FOXP3+ cell analysis from the draining cervical lymph nodes (CLN; top) and spinal cords (bottom) of mice injected with EB-NPCs, fibroblasts, or HBSS. (**B**) Quantification of the number of CD4+FOXP3+ Tregs demonstrated a significant (p < 0.05) increase in the CLNs of EB-NPC transplanted mice compared to controls at day 5 p.t. (top). An increase in Tregs was not detected in the CLNs or spinal cords of EB-NPC transplanted mice by day 7 p.t. (bottom). Data represents two independent experiments. For day 5 analysis, n = 4 (EB-NPC), n = 6 (Fibroblast), and n = 4 (HBSS) animals per group. For day 7 analysis, n = 6 (EB-NPC), n = 7 (Fibroblast), and n = 5 (HBSS) animals per group. Data is presented as average ± SEM and was analyzed using one-way ANOVA followed by Tukey’s multiple comparison test.

CD4+/FOXP3+ Tregs are a subset of suppressive T cells that are crucial to the development and maintenance of self-tolerance that have been shown to prevent autoimmune disease [26]. JHMV-infected animals exhibit dampened neuroinflammation and attenuated disease pathology as a result of Treg adoptive transfer [27]. We have previously shown that Tregs induced in the CNS following transplantation of DD-NPCs [14] were critical for clinical and histologic improvement in mice persistently infected with JHMV. We evaluated the levels of CD4+/FOXP3+ Tregs in the draining cervical lymph nodes (CLNs) and CNS of animals transplanted with EB-NPCs at days 5, 7, and 21 p.t. Tregs were significantly increased in the CLNs, but not the CNS, of EB-NPC transplanted animals compared to fibroblast and HBSS injected controls at day 5 p.t. (Fig 5A). Increased Treg accumulation in the CLNs and spinal cord was not detected in EB-NPC, fibroblast, or HBSS injected animals at days 7 and 21 p.t., suggesting a rapid and transient induction of Tregs in EB-NPC-transplanted mice (Fig 5B **and data not shown**).

**Fig 6:**
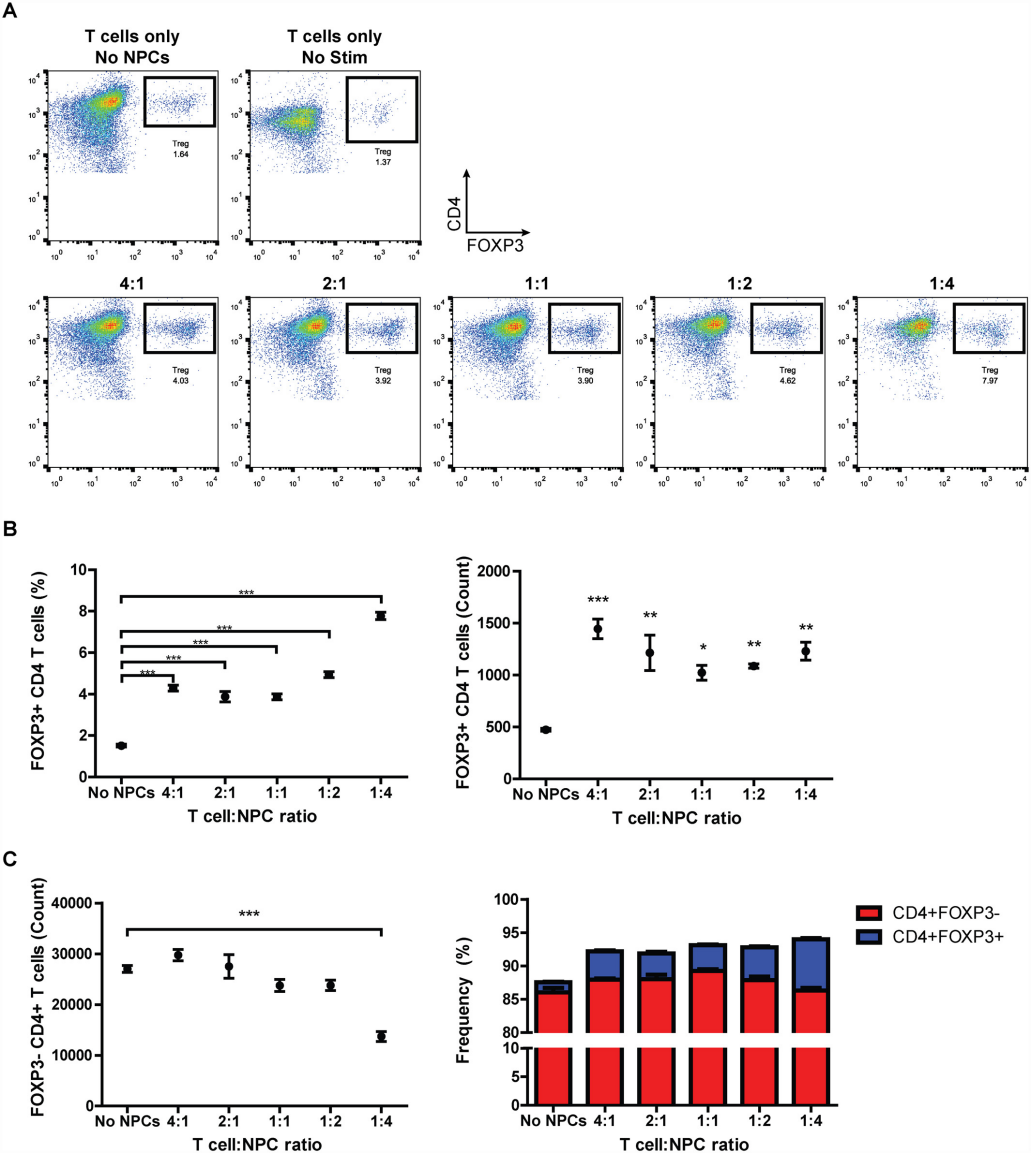
Human iPSC-derived NPCs directly induce Treg conversion *in vitro*. (**A**) Representative FACS plots of CD4+FOXP3+ Tregs from EB-NPC-T cell co-cultures. T cells were mixed with EB-NPCs at varying T cell-to-EB-NPC ratios and activated in the presence of anti-CD3/anti-CD28 beads for three days. (**B**) The frequency and number of FOXP3-expressing CD4+ T cells was significantly increased in the presence of EB-NPCs. (**C**) The observed increase in Tregs was correlated with a significant decrease in the number of conventional CD4+ T cells at a T cell-to-EB-NPC ratio of 1:4. All data was analyzed using students t-test and is presented as average ± SEM; *p < 0.05; **p < 0.01; ***p < 0.001; n = 4.

To determine if EB-NPCs could directly convert T cells to a Treg phenotype, EB-NPCs were co-cultured with T cells enriched from splenoctyes of non-infected mice in culture conditions that support T cell survival and activation. After 3 days, an increase in the frequency and number of CD4+FOXP3+ Tregs was observed in cultures when activated T cells were co-cultured with EB-NPCs, but not when T cells were cultured in the absence of EB-NPCs (Fig 6A and 6B). Furthermore, Treg induction was proportionate to the number of EB-NPCs and was concomitant with a decrease in conventional CD4+FOXP3- T cells (Fig 6C). We previously demonstrated that *in vitro* Treg induction by NPCs produced by another method was dependent on secretion of the pleiotropic cytokine transforming growth factor-beta (TGF-β) [14]. However, compared to DD-NPCs, secretion of active TGF-β1 or TGF-β2 could not be detected in the culture media of EB-NPCs (S4 Fig).

**Fig 7:**
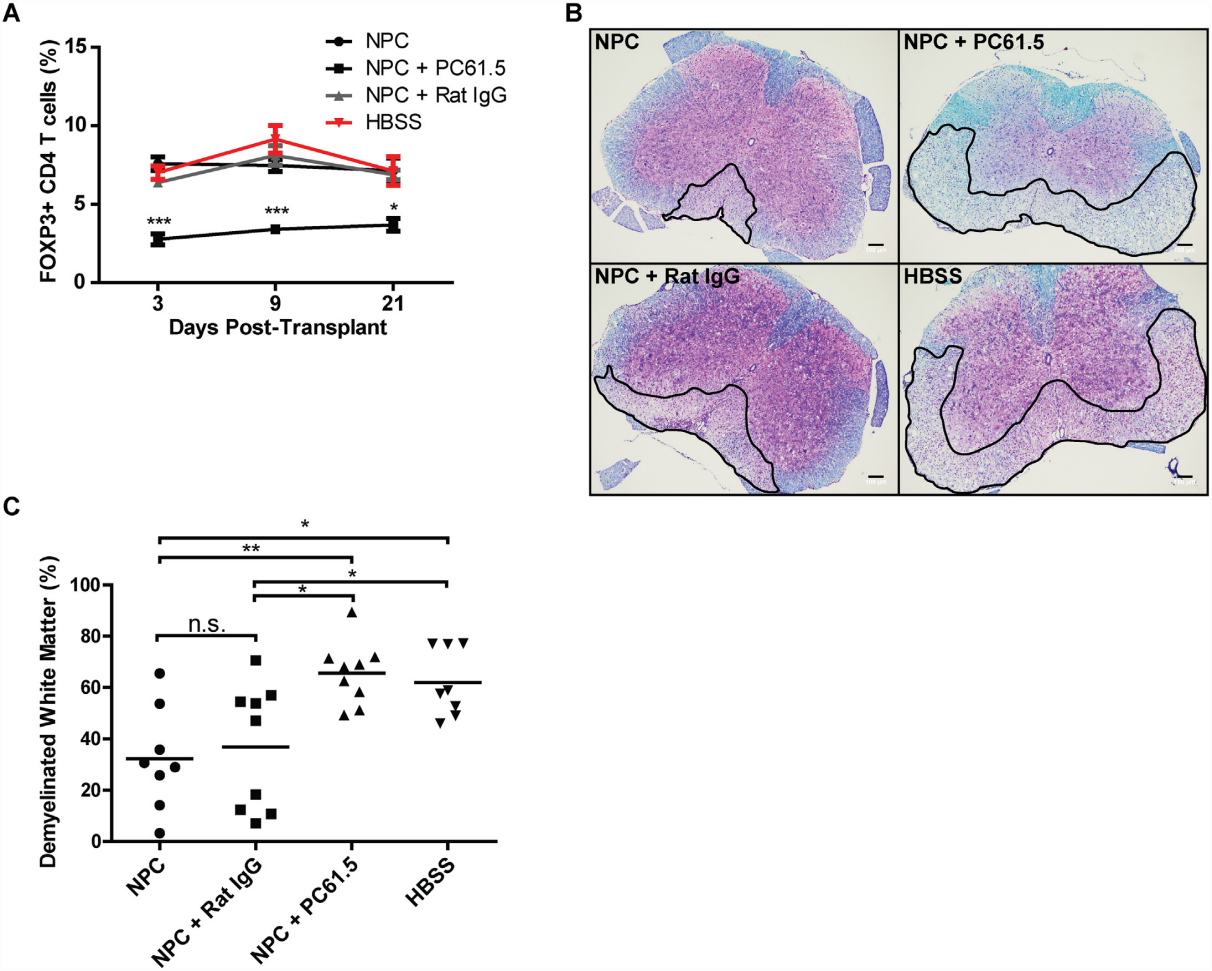
Regulatory T cells are necessary for EB-NPC-induced myelin sparing. (**A**) Treatment of EB-NPC-transplanted mice with PC61.5, a rat monoclonal antibody raised against CD25, resulted in a significant reduction in the frequency of circulating CD4+FOXP3+ cells that was sustained to day 21 p.t. (**B**) Representative spinal cord sections stained with LFB and H&E. Outlined areas highlight demyelination. (**C**) Quantification of white matter damage revealed PC61.5-treated EB-NPC-transplanted mice did not have reduced demyelination when compared to non-treated EB-NPC-transplanted mice. For (***A***) and (***C***), data represents two independent experiments with n = 8 (EB-NPC alone), n = 9 (EB-NPC + IgG control), n = 9 (EB-NPC + PC61.5), and n = 8 (HBSS) animals per group. All data is presented as the average ± SEM and was analyzed using one-way ANOVA followed by Tukey’s multiple comparison test; *p < 0.05, **p < 0.01, ***p < 0.001.

Treatment with the monoclonal antibody PC61.5 (anti-murine CD25 rat IgG1) depletes CD4+FoxP3+ Tregs and is widely used to characterize Treg function in vivo [20, 21]. In order to evaluate the requirement of Tregs for remyelination following EB-NPC transplantation, mice were injected with PC61.5 at days -2, 0, and 2 p.t. Treatment with anti-CD25 resulted in a decrease in the frequency of circulating Tregs by day 3 p.t. that was sustained out to day 21 p.t., the day spinal cords were collected for histology (Fig 7A and 7B). *In vivo* ablation of Tregs resulted in increased demyelination compared to non-treated EB-NPC transplanted animals (Fig 7C and 7D).

### EB-NPCs are distinct cell type compared to our previously published DD-NPCs

**Fig 8:**
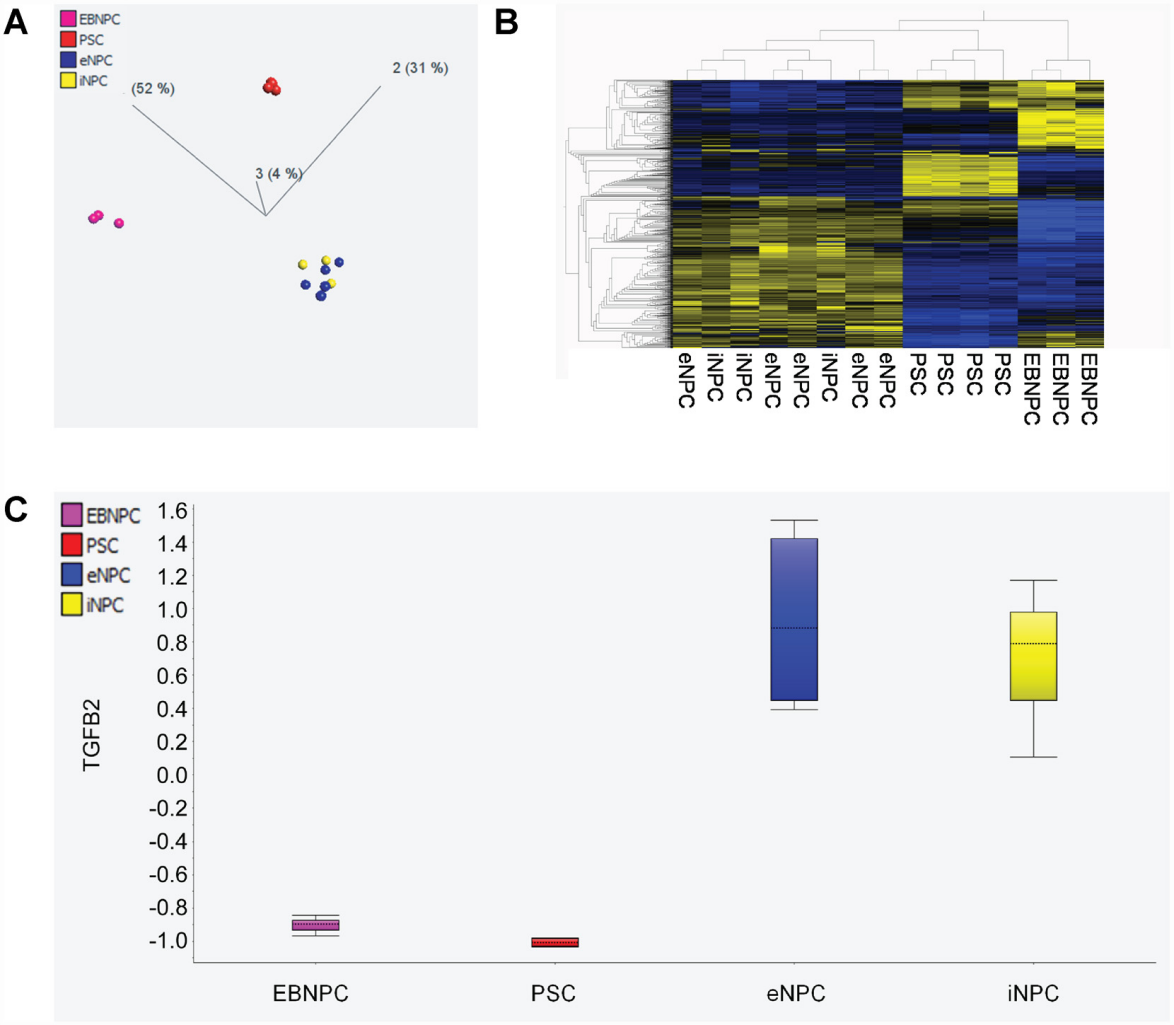
EB-NPCs are significantly different than our previously differentiated NPCs. EB-NPCs cluster away from both pluripotent stem cells and NPCs derived from both ESCs and iPSCs by our previously published method in both (**A**) Principal component analysis and (**B**) hierarchical clustering. (**C**) EB-NPCs show a lack of TGFβ2 transcript as shown by whole genome expression analysis.

Although EB-NPCs shared many characteristics with previously published DD-NPCs, they were unable to promote significant clinical recovery in transplanted mice. In order to elucidate these differences, we employed whole genome expression analysis and compared EB-NPCs with DD-NPCs produced using the previously reported protocol [14]. Principal component analysis of all genes with a variance of 0.3 or greater was used to examine the relationships among various preparations of cells (Fig 8A). Four groups of cells were analyzed, including pluripotent stem cells (PSCs), EB-NPCs, and DD-NPCs derived from both ESCs and iPSCs (eDD-NPCs and iDD-NPCs respectively). This analysis demonstrated that DD-NPCs from iPSCs and ESCs comingled as one group, while undifferentiated PSCs and EB-NPCs had distinct gene expression profiles. This result was also observed a hierarchically-sorted heat map of the samples was examined (Fig 8B). We subsequently inspected specific genes previously identified as upregulated in DD-NPCs to determine their expression levels in EB-NPCs. In particular, we previously reported that TGF-β2 expression was upregulated in DD-NPCs and may be critical for clinical recovery in the JHMV model of demyelination [14]. DD-NPCs derived from both iPSCs and hESCs expressed high levels of TGF-β2, while EB-NPCs and undifferentiated PSCs did not, consistent with our ELISA data (Fig 8C **and** S4 Fig).

## Discussion

Neural precursor cells (NPCs) are rapidly transitioning to the clinic for the treatment of a variety of neurological disorders. Human trials testing safety and efficacy of fetal- or hESC-derived NPC grafts in patients with chronic spinal cord injury, amyotrophic lateral sclerosis, and Pelizaeus-Merzbacher disease are currently being performed [28, 29]. Given that current FDA-approved front-line therapies do not offer relief from symptoms for patients with progressive forms of disease, MS is an attractive target for cell-based therapeutics. However, the great diversity of NPCs, whose characteristics are determined by their sources, methods of derivation and culture conditions, warrants thorough investigation of the therapeutic value of specific NPC types [15].

Human NPCs isolated from fetal CNS tissue have an extensive history of transplantation in animal models of neurodegenerative disease. Non-human primates afflicted with autoimmune encephalomyelitis (EAE) demonstrated improvements in disease pathology and clinical outcome when human fetal NPCs were injected systemically [11]. However, long-term immune suppression via administration of the calcineurin inhibitor cyclosporine was required to prolong the survival of fetal NPCs *in vivo*. Evidence suggests allogeneic NPCs are rapidly rejected following transplantation into immune competent animals with ongoing neuroinflammation [30], and both the immunomodulatory and trophic support functions of fetal NPCs have been shown to be limited temporally [31, 32]. Moreover, the use of fetal-derived NPCs for transplantation may be restricted by the consistency and quality of donor tissue.

Human pluripotent stem cells (hPSCs) have the intrinsic capacity for unlimited self-renewal and for the production of differentiated cell types suitable for transplantation therapy. NPCs derived from hESCs ameliorate the clinical course of EAE by modulation of peripheral T cells in affected mice [10]. However, as with fetal NPCs, the neuroprotective effect of transplanted hESC-NPCs in EAE was dependent on survival and migration of the xenograft. In contrast, we recently demonstrated that sustained clinical recovery could be achieved in spite of xenograft rejection following intraspinal injection of NPCs directly differentiated from hPSCs (DD-NPCs) in a virus-induced model of neuroinflammatory demyelination [14]. Suppression of lymphocyte infiltration was facilitated by local induction of Tregs, contradicting previous findings that hESC-NPCs must migrate to the peripheral lymphatic system and inhibit dendritic cell maturation or T cell activation in the lymph nodes for histologic improvement to be observed [10].

Here, we report the first evaluation of the therapeutic potential of human NPCs derived by an embryoid body (EB)-based method in a model of virus-induced immune-mediated demyelination. We implemented a FACS-based strategy to enrich for a population of NPCs free of glial and neural crest contaminants. FACS-sorted NPCs expressed hallmark markers of neural stem and precursor cells and subsequently differentiated to the major CNS lineages *in vitro*. As with DD-NPCs, EB-NPCs were rapidly rejected following injection into the spinal cords of immunocompetent JHMV-infected mice, and no cellular migration to peripheral immune organs was observed. In spite of rejection, CD4+ T cell infiltration was muted in the CNS of EB-NPC-transplanted mice, which correlated with a transient induction of Tregs in the draining CLNs. Tregs, a T cell lineage essential to the establishment and maintenance of self-tolerance, have been shown to be protective in animal models of inflammatory and autoimmune disease, including EAE and JHMV-induced demyelination [27, 33]. Furthermore, NPCs have been suggested to convert conventional and encephalitogenic T cells to a Treg phenotype [34, 35], and DD-NPC-induced recovery in JHMV-infected animals was dependent on local Treg induction [14]. Indeed, EB-NPCs induced conventional naïve T cells to differentiate toward a Treg phenotype *in vitro*, and *in vivo* ablation of Tregs by monoclonal antibody blockade abrogated histopathological improvement in transplanted mice. Induction of Tregs and dampened accumulation of CD4+ effector T cells was accompanied by remyelination in the white matter tracts of EB-NPC injected animals. Remyelination was evaluated at the point at which EB-NPCs could no longer be detected in the CNS by immunohistochemical staining or *in vivo* imaging, suggesting that endogenous myelin regeneration, and not cell replacement by EB-NPCs, was responsible for the observed histologic improvement. Xenograft rejection has been proposed to foster limited remyelination following transplantation of human glial-restricted precursors in the JHMV model [36]. In this study, we did not observe enhanced remyelination or a reduction in infiltrating lymphocytes in animals transplanted with human fetal fibroblasts, confirming the specificity of EB-NPC-induced remyelination. Thus, xeno-antigens alone are not sufficient to promote the focal remyelination induced by EB-NPCs.

In contrast to our previous study using DD-derived NPCs, JHMV-infected mice transplanted with EB-NPCs did not display significant improvements in clinical outcome. There are several hypotheses that may explain observed differences in clinical improvement in our model, and of particular interest is the production of transforming growth factor beta (TGF-β) by NPCs. TGF-β is a ubiquitous cytokine that exists in at least three isoforms: TGF-β1, TGF-β2, and TGF-β3. Generation of induced Tregs from CD4+ precursors requires stimulation of the TGF-β receptor, and TGF-β2 triggers FOXP3 expression in conventional CD4+ T cells [37]. Using transcriptome analysis, Chen et al. (2014) observed upregulation of TGF-β1 and TGF-β2 in DD-NPCs, and secreted TGF-β was detected in DD-NPC supernatants. Immunoassay of media collected from EB-NPC cultures did not detect TGF-β1 or TGF-β2, which may explain the different spatial and temporal dynamics of Treg induction, and lack of functional improvement, in EB-NPC-transplanted JHMV-infected mice. An additional pleiotropic cytokine that may contribute to immune modulation and repair in models of demyelination is leukemia inhibitory factor (LIF). LIF promotes Treg induction mainly by suppressing differentiation of conventional CD4+ T cells to a pro-inflammatory phenotype [38, 39]. Moreover, LIF is secreted by transplanted murine NPCs and is proposed to be involved in NPC-induced neuroprotection in EAE [40, 41]. Similar to TGF-β, human LIF could not be detected in EB-NPC supernatants (data not shown). Thus, it is likely that the methods used to generate and isolate EB-NPCs in this study may have yielded a population of cells that lack the appropriate characteristics to confer significant and sustained repair in JHMV-infected mice.

## Conclusion

This report highlights the need for rigorous characterization and selection of therapeutically valuable cell types derived from human pluripotent stem cells for the treatment of neurodegenerative disease. The ability to suppress neuroinflammation via Treg induction and promotion of endogenous remyelination makes NPCs an attractive candidate therapeutic for treating MS. However, the use of NPCs for cell based therapies is complicated by the growing number of methods to generate neuroectodermal-lineage cell types from hESCs and hiPSCs [42]. When significant clinical improvement was observed following transplantation of DD-NPCs into JHMV-infected mice, cells were selected for intraspinal transplantation based on a definitive transcriptomic signature [14]. In-depth methods for validating the genetic and functional phenotype of clinically relevant NPCs, as well as standardized protocols for driving pluripotent stem cells to generate disease-modifying NPCs, will be critical for future success of NPC transplantation in patients with neurologic disorders.

## Supporting Information

S1 Fig Microscopy of EB-NPCs confirms expression of neural lineage markers. FACS-sorted EB-NPCs expressed markers characteristic of NPCs, including (**A**) NESTIN, (**B**) SOX2, and (**C**) PAX6. (**D-G**) Differentiated EB-NPCs expressed markers restricted to neurons (Tuj1+) astrocytes (GFAP)+ astrocytes, and the oligodendrocyte lineage (NG2 & Olig2). Scale bars = 100 μm.
(TIF)

S2 Fig Intraspinal delivery of EB-NPCs does not promote neurologic recovery in mice persistently infected with JHMV. JHMV-infected mice were transplanted with either EB-NPCs, fibroblasts, or HBSS and clinical disease recorded following spinal cord injection. Graph of clinical scores of mice injected intraspinally with EB-NPCs (black), human fibroblasts (red), and HBSS (blue) at defined time points post-transplant (p.t.). No improvement in locomotion was observed by day 21 p.t. Clinical evaluation was based on the following scoring system: 0, asymptomatic; 0.5, ruffled fur; 1, limp tail; 2, waddling gait without righting difficulty; 2.5, waddling gait accompanied by righting difficulty; 3, hind-limb weakness and extreme righting difficulty; 3.5, complete hind limb paralysis; and 4, death. Data represents two independent experiments and is presented as average ± SEM.
(TIF)

S3 Fig Spinal cord accumulation of macrophages, microglia, and virus-specific T cells is unaffected by EB-NPC transplantation. (**A**) Representative FACS plots demonstrating gating strategies for macrophages (CD45^hi^, F4/80^+^), microglia (CD45^lo^, F4/80^+^), and T cells specific for the CD4 immunodominant epitope M133–147 or the CD8 immundominant epitope S510-518. (**B**) Quantification of the frequencies of infiltrating macrophages, microglia, M133-147+ CD4 T cells, and S510-518+ CD8 T cells reveals no difference between EB-NPC, fibroblast, and HBSS injected animals. Data is presented as average ± SEM and represents 3 animals per treatment group.
(TIF)

S4 Fig Secreted TGF-β is detected in hESC-NPC, but not EB-NPC, culture media. Enzyme linked immunosorbent assay (ELISA) results demonstrating levels of TGF-β1 and TGF-β2 in culture media collected from hESC-derived NPCs and hiPSC-derived NPCs; n.d. = not detected. Data is presented as average ± SEM and represents 3 independent experiments.
(TIF)
