# Supplementary figures and images for: Remyelination Is Correlated with Regulatory T Cell Induction Following Human Embryoid Body-Derived Neural Precursor Cell Transplantation in a Viral Model of Multiple Sclerosis
Source: PLoS One. 2016 Jun 16;11(6):e0157620. doi: 10.1371/journal.pone.0157620 (PMC4911106; doi:10.1371/journal.pone.0157620)

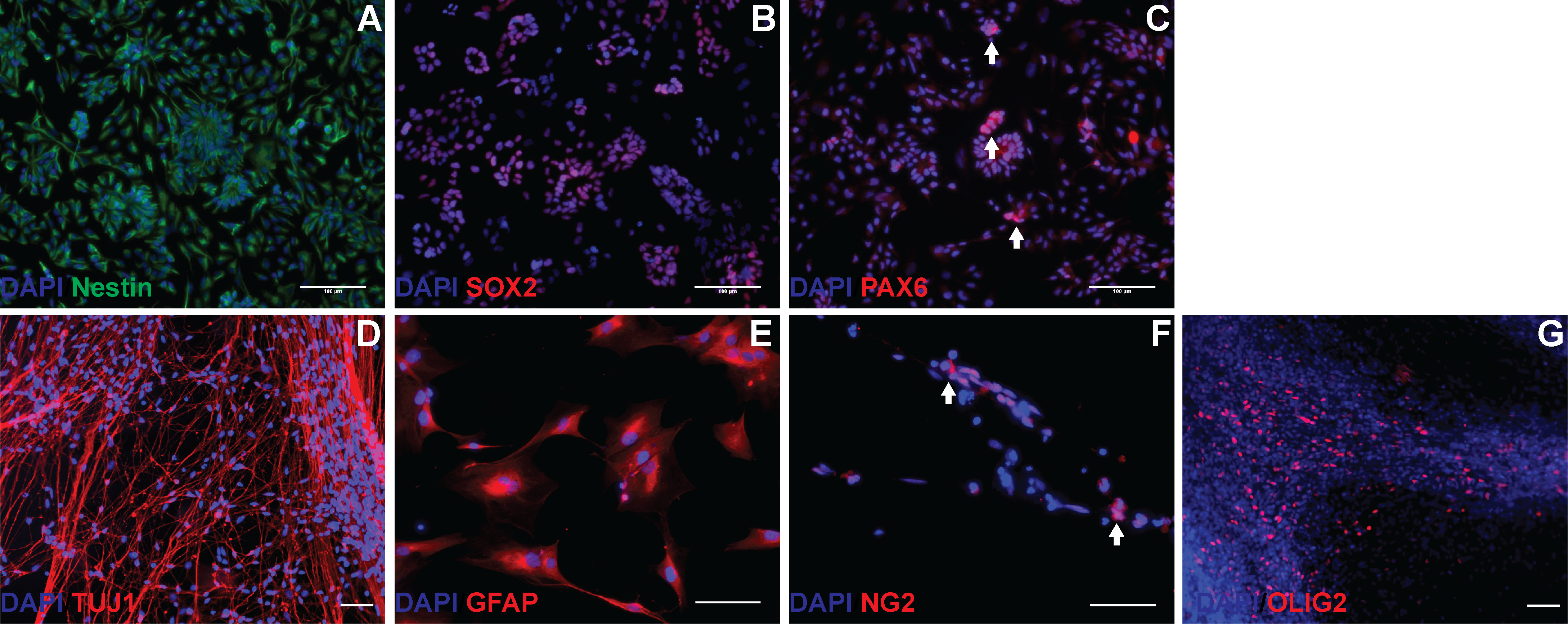

Supplement: S1 Fig — FACS-sorted EB-NPCs expressed markers characteristic of NPCs, including (A) NESTIN, (B) SOX2, and (C) PAX6. (D-G) Differentiated EB-NPCs expressed markers restricted to neurons (Tuj1+) astrocytes (GFAP)+ astrocytes, and the oligodendrocyte lineage (NG2 & Olig2). Scale bars = 100 μm. (TIF) [file pone.0157620.s001.tif]

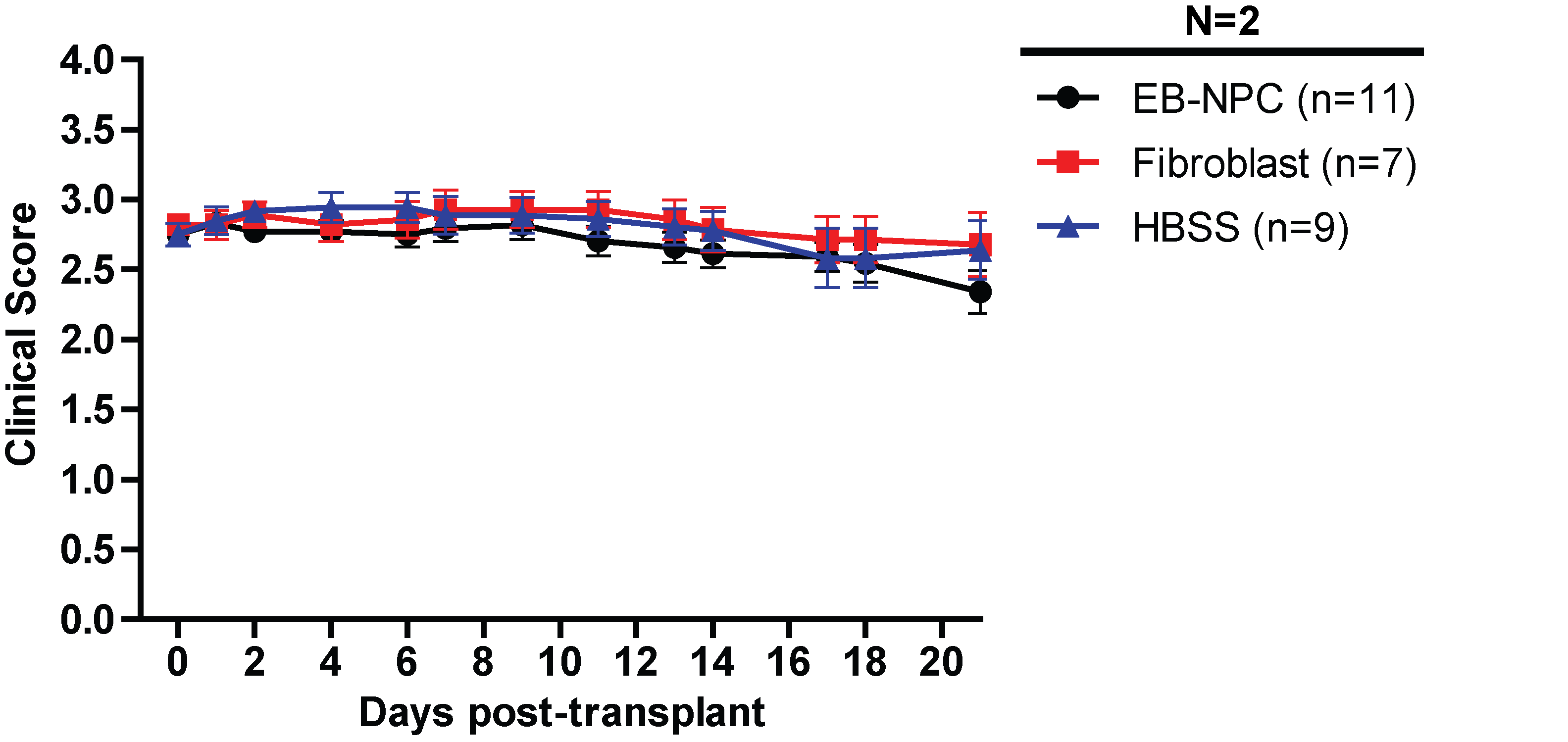

Supplement: S2 Fig — JHMV-infected mice were transplanted with either EB-NPCs, fibroblasts, or HBSS and clinical disease recorded following spinal cord injection. Graph of clinical scores of mice injected intraspinally with EB-NPCs (black), human fibroblasts (red), and HBSS (blue) at defined time points post-transplant (p.t.). No improvement in locomotion was observed by day 21 p.t. Clinical evaluation was based on the following scoring system: 0, asymptomatic; 0.5, ruffled fur; 1, limp tail; 2, waddling gait without righting difficulty; 2.5, waddling gait accompanied by righting difficulty; 3, hind-limb weakness and extreme righting difficulty; 3.5, complete hind limb paralysis; and 4, death. Data represents two independent experiments and is presented as average ± SEM. (TIF) [file pone.0157620.s002.tif]

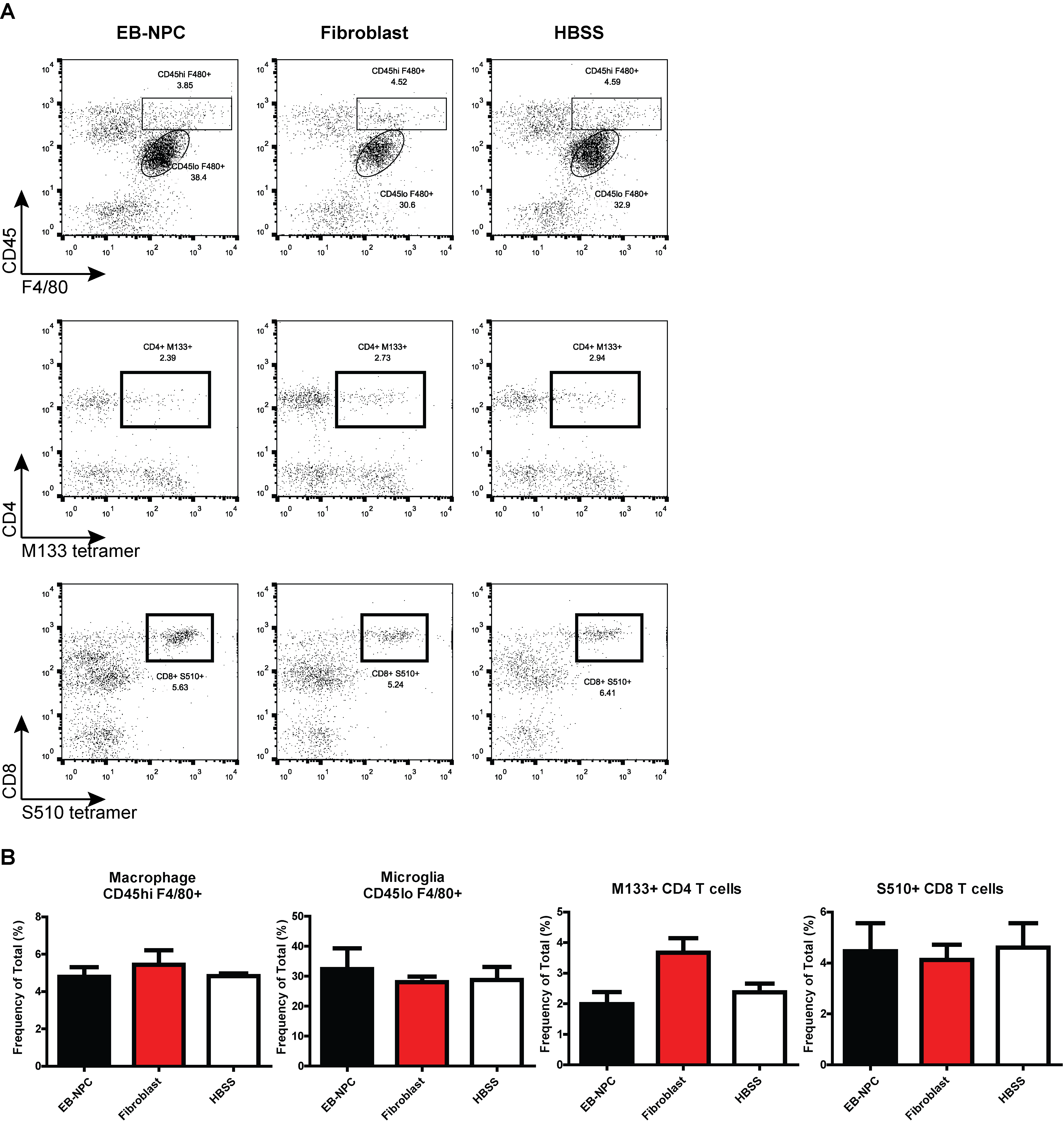

Supplement: S3 Fig — (A) Representative FACS plots demonstrating gating strategies for macrophages (CD45hi, F4/80+), microglia (CD45lo, F4/80+), and T cells specific for the CD4 immunodominant epitope M133–147 or the CD8 immundominant epitope S510-518. (B) Quantification of the frequencies of infiltrating macrophages, microglia, M133-147+ CD4 T cells, and S510-518+ CD8 T cells reveals no difference between EB-NPC, fibroblast, and HBSS injected animals. Data is presented as average ± SEM and represents 3 animals per treatment group. (TIF) [file pone.0157620.s003.tif]

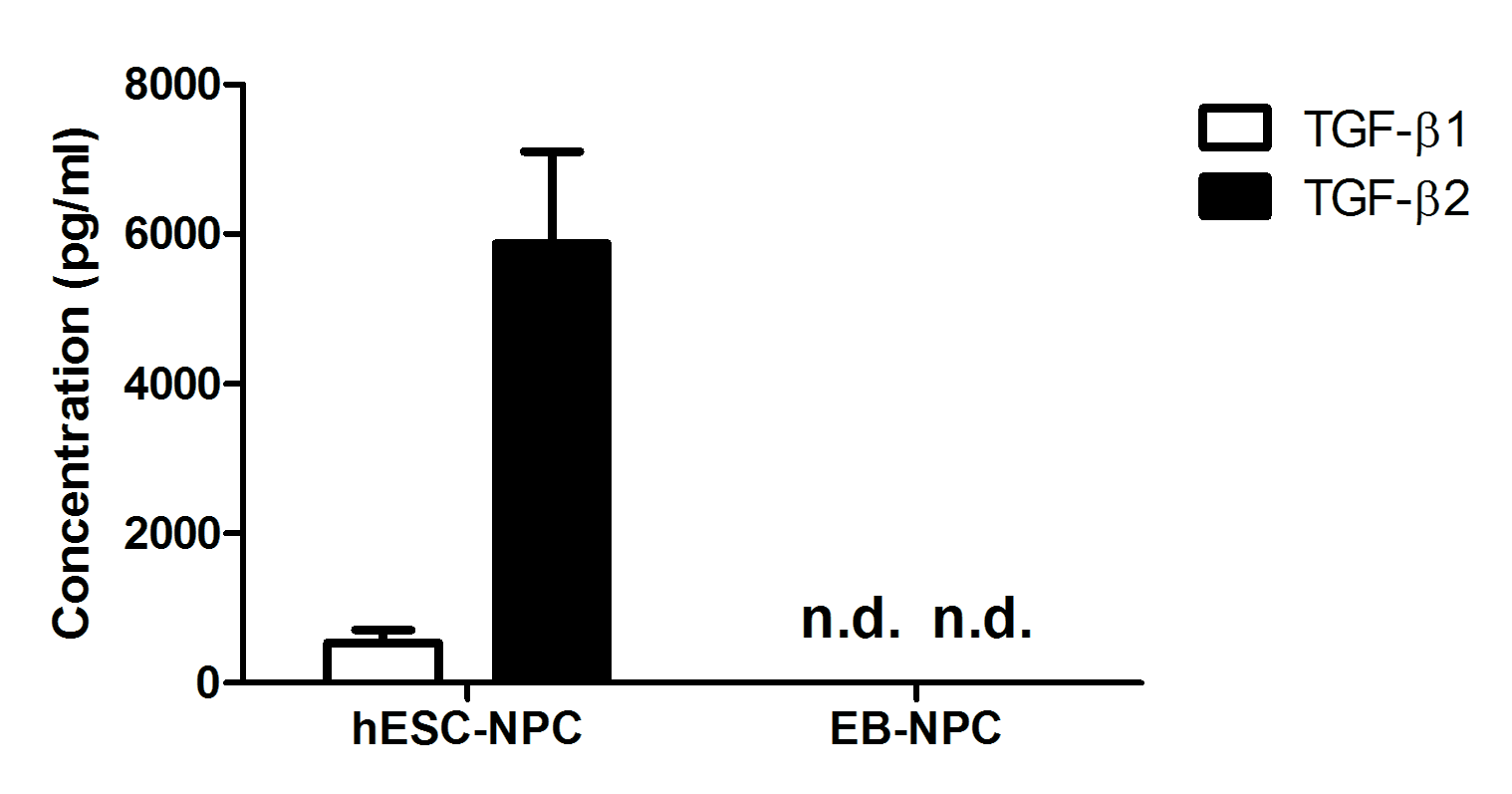

Supplement: S4 Fig — Enzyme linked immunosorbent assay (ELISA) results demonstrating levels of TGF-β1 and TGF-β2 in culture media collected from hESC-derived NPCs and hiPSC-derived NPCs; n.d. = not detected. Data is presented as average ± SEM and represents 3 independent experiments. (TIF) [file pone.0157620.s004.tif]
